# The Impact of Gene Dosage and Heterozygosity on the Diploid Pathobiont *Candida albicans*

**DOI:** 10.3390/jof6010010

**Published:** 2019-12-27

**Authors:** Shen-Huan Liang, Richard J. Bennett

**Affiliations:** Department of Molecular Microbiology and Immunology, Brown University, Providence, RI 02912, USA; shen-huan_liang@brown.edu

**Keywords:** hemizygosity, loss of heterozygosity (LOH), commensalism, virulence, aneuploidy, mitotic recombination, parasexual cycle, drug resistance

## Abstract

*Candida albicans* is a fungal species that can colonize multiple niches in the human host where it can grow either as a commensal or as an opportunistic pathogen. The genome of *C. albicans* has long been of considerable interest, given that it is highly plastic and can undergo a wide variety of alterations. These changes play a fundamental role in determining *C. albicans* traits and have been shown to enable adaptation both to the host and to antifungal drugs. *C. albicans* isolates contain a heterozygous diploid genome that displays variation from the level of single nucleotides to largescale rearrangements and aneuploidy. The heterozygous nature of the genome is now increasingly recognized as being central to *C. albicans* biology, as the relative fitness of isolates has been shown to correlate with higher levels of overall heterozygosity. Moreover, loss of heterozygosity (LOH) events can arise frequently, either at single polymorphisms or at a chromosomal level, and both can alter the behavior of *C. albicans* cells during infection or can modulate drug resistance. In this review, we examine genome plasticity in this pathobiont focusing on how gene dosage variation and loss of heterozygosity events can arise and how these modulate *C. albicans* behavior.

## 1. Introduction

The genome of a species is not constant but fluctuates due to both neutral events and those shaped by natural selection. A wide variety of processes introduce genetic diversity into a population, from those that act at the level of single nucleotides (e.g., de novo base substitutions) to those leading to chromosome-level changes (e.g., the acquisition of supernumerary chromosomes producing aneuploid forms). Each of these changes has the potential to alter cellular behavior, and thus knowledge of the totality of changes impacting a genome is essential for understanding of a species. 

**Figure 1:**
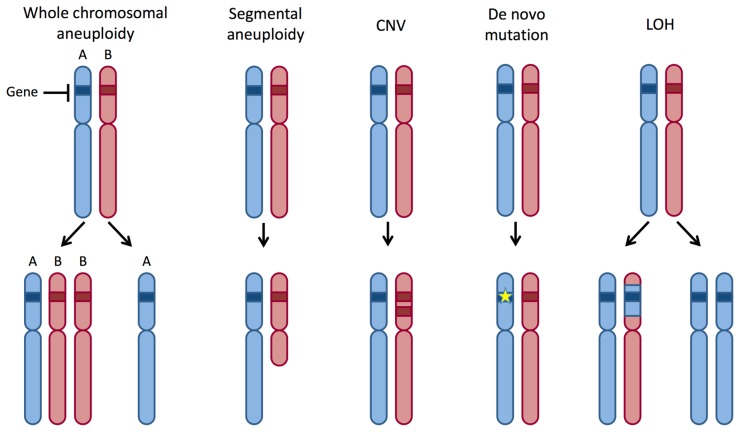
Genetic mechanisms that can impact gene dosage. In a heterozygous diploid strain, each chromosome has two homologs, A and B. Whole chromosomal aneuploidy is a process in which cells gain or lose whole chromosomes. Segmental aneuploidy refers to a gain or loss of a substantial region of a chromosome. Copy number variants (CNVs) arise when the number of copies of a particular gene differs between strains. De novo mutations are mutations that arise within a genome and include single nucleotide polymorphisms (SNPs) and insertions/deletions (indels) that could change gene activity. Loss of heterozygosity (LOH) is a process in which genetic information is lost from one of the two chromosome homologs and can impact either parts of a chromosome or involve whole chromosomes.

A frequent cause of genomic variation is due to changes in gene dosage. These can occur due to copy number variants (CNVs) arising from insertions, deletions or amplifications of segments of the genome (Figure 1). Expression profiling reveals that the gene copy number often, but not always, correlates with gene expression in yeast, mammals and plants [1,2,3,4], with notable exceptions being animal sex chromosomes where dosage compensation mechanisms may apply [5]. Changes in gene dosage can also occur on a chromosomal scale due to aneuploidy, where an abnormal complement of chromosomes is present in the cell (Figure 1). Gene dosage variation can drive genome evolution, but is also associated with sporadic and Mendelian diseases in humans [6].

Diploid genomes can be heterozygous in which polymorphisms distinguish the two chromosome homologs. This can impact cellular traits when allelic differences result in altered gene expression or in gene products with disparate activities. In addition, differences between the two alleles of a gene can result in haploinsufficiency, where a single allele is insufficient to support normal gene function. 

Haploinsufficiency is associated with human diseases including autism spectrum disorders and schizophrenia [7], and can contribute to cancer by impacting tumor-suppressor genes [8]. While ~300 human genes have been shown to exhibit haploinsufficiency [9], this is likely an underestimation, given that up to 20% of *Saccharomyces cerevisiae* genes exhibit a haploinsufficient phenotype [10], and over half of essential *S. cerevisiae* genes display haploinsufficiency under some conditions [11]. 

In general, it is now clear that even relatively small changes in gene expression can have a profound effect on cell behavior. For example, in haploinsufficiency, a twofold drop in protein levels may be sufficient to compromise normal gene function. This can be due to an imbalance in the stoichiometry of subunits required for protein complexes [12], and in line with this, protein components of macromolecular complexes are often expressed at precisely the levels necessary for correct stoichiometry [13]. Moreover, *S. cerevisiae* genes with low heterozygote fitness tend to be those that encode the subcomponents of protein complexes [14]. Many genes have therefore evolved to maintain expression levels within a narrow window that avoids under- or overexpression, as either may have negative consequences on fitness [15].

In this review, we highlight how genomic plasticity influences the biology of the diploid fungus *Candida albicans*, focusing on the impact of heterozygosity and gene dosage on phenotypic traits. This species is a prevalent commensal, able to colonize multiple niches in the human body including the gastrointestinal and reproductive tracts of most healthy adults. However, it is also an opportunistic pathogen capable of causing a wide variety of mucosal and disseminated infections, the latter associated with a mortality rate of ~40% in adults [16]. There is now an increasing appreciation for how genomic alterations in *C. albicans* can alter its phenotypic properties in vitro, as well as its commensal and pathogenic properties in murine models of infection. Here, we emphasize how genetic changes contribute to phenotypic diversity and promote adaption both to host niches and to antifungal drugs.

## 2. The Heterozygous Diploid Genome of *C. albicans*

*C. albicans* isolates are naturally diploid and contain eight heterozygous chromosomes [17,18,19]. Whole genome sequencing revealed that isolates have, on average, one heterozygous SNP every 200–300 bp, although heterozygosity levels can vary substantially from isolate to isolate [20,21]. Differences in heterozygosity are often due to loss of heterozygosity (LOH) events, where genetic information from one of the two chromosomal homologs has been lost. LOH can be limited in scope or can extend across large regions of the chromosome, or even affect whole chromosomes (Figure 1) [19,20,21]. Most large, segmental LOH events (>50 kb) reflect break-induced replication (BIR) or reciprocal crossover events, as they map to chromosomal regions that extend from the recombination site to the chromosome end [22]. 

Analysis of clinical isolates reveals that the population structure is largely clonal with 17 distinct clades [21]. Interestingly, heterozygosity levels are lower in clade 13 isolates than in other clades, and these isolates (also called *Candida africana*) appear to be limited to causing genital tract infections [21]. In line with this, this clade has undergone extensive ancestral LOH events and fixed mutations that inactivate genes associated with systemic infection [21]. *C. dubliniensis* is a closely related sister species to *C. albicans* that also appears to have undergone massive LOH and acquired gene-disrupting mutations that may contribute to its lower fitness and reduced virulence [21]. Moreover, a comparative analysis of 21 clinical isolates of *C. albicans* [20] revealed that higher levels of genome heterozygosity correlate with faster growth rates in replete medium, establishing a link between overall heterozygosity levels and fitness. 

In SC5314, the standard laboratory isolate of *C. albicans*, ~200 genes contain heterozygous single nucleotide polymorphisms (SNPs) that cause premature stop codons [23], indicating that a number of genes are likely to be functionally heterozygous. Furthermore, more than half of open reading frames contain SNPs, the vast majority of which result in a change in the protein sequence [23], and one such SNP was shown to inactivate an allele of *HIS4* in SC5314 [24]. Muzzey et al. demonstrated that allelic-specific effects regulate RNA expression levels genome wide [25]. For example, allele B of orf19.238 has a nearly twofold higher RNA expression over allele A, while orf19.3556 has a 30% RNA expression bias between its two alleles. Staib et al. similarly showed that the two alleles of *SAP2* are differentially expressed due to differences in pentameric repeats in their promoters [26]. This parallels studies where tandem repeat regions in the promoters of isolates from two *Aspergillus* species correlated with the tuning of gene expression [27]. Allele-expression differences are not limited to RNA levels, as ribosome profiling revealed that ~4% of *C. albicans* alleles also exhibit differences in translation [28]. Allelic differences therefore contribute to both transcriptional and translational efficiencies in *C. albicans*, and have the potential to significantly influence multiple phenotypic traits. 

The fact that different alleles can encode for proteins with different functions has been well documented for the *C. albicans* ALS (Agglutin-Like Sequence) family of genes. This family encodes cell surface glycoproteins implicated in adhesion to a wide variety of host ligands [29]. Allelic diversity within *ALS* genes is largely due to differences in the number of tandem repeats of a central 108 bp motif. For example, in SC5314, the two alleles of *ALS3* include one allele with 12 tandem repeats and one allele with 9 tandem repeats, and the longer allele encodes for a protein with greater adhesion to host cells than that produced by the shorter allele [30]. Analysis of *ALS3* alleles from diverse isolates revealed considerable diversity in the number of tandem repeats, from 6 to 19, and that most strains contained both a longer and a shorter allele, suggesting that differences in size may be selected for [30]. Diversity between the alleles of other *ALS* family genes is also apparent, with high allelic diversity linked to recombination between tandem repeats, as well as differences due to mutations within the more conserved domains flanking the tandem repeats [30,31,32].

On a broader note, the *C. albicans* genome contains more protein-encoding genes that contain short repeats than other fungi, and thus there is considerable potential for these genes to diversify by an extension or contraction of these repeats [33,34]. Some genes have acquired synonymous mutations that decrease the likelihood that recombination will change the number of repeats, while selection may also operate to optimize the precise number of tandem repeats associated with a particular strain background or a specific host niche [34]. 

## 3. Mating, Ploidy Shifts and Aneuploidy

Despite a largely clonal population structure, evidence exists for recombination between *C. albicans* isolates based on the analysis of both nuclear and mitochondrial genomes [21,22]. This is consistent with a functional sexual or parasexual cycle operating in the species [35,36]. Most *C. albicans* isolates are heterozygous at the mating-type-like (*MTL*) locus on chromosome (Chr) 5, and thus contain both *MTL***a** and *MTL*α idiomorphs [37,38]. The **a** and α loci contain four transcription factors that regulate mating and cell identity (**a**1, **a**2 and α1, α2, respectively) as well as three ‘nonsex’ genes, *PIK*, *PAP* and *OBP* [37]. Deletion of a single *MTL* locus, the nonsex genes from one *MTL* locus, or just the two *OBP* alleles, causes a modest reduction in virulence in a murine model of systemic infection [39]. Ibrahim et al. also found that generating *MTL* homozygous strains by growth on sorbose medium could, at least in a subset of cases, result in a decrease in virulence [40]. However, while growth on sorbose can promote the homozygosis of Chr 5 (see below), it can also cause additional genomic changes [41] that complicate the analysis of sorbose-selected isolates.

*MTL* homozygous **a** and α cells readily undergo phenotypic switching between two cellular states, white and opaque [38,42]. White cells are essentially sterile whereas opaque cells are mating competent, and the white-to-opaque transition is restricted by the **a**1/α2 complex present in *MTL***a**/α cells [43]. Conjugation of opaque **a** and α cells (heterothallic mating) generates tetraploid **a**/α cells that are less virulent and less competitive than diploid cells in a model of systemic infection [40]. Recent profiling studies revealed few transcriptional differences between *C. albicans* diploid and tetraploid cells [44], similar to the analysis of *S. cerevisiae* cells of differing ploidy [45,46].

No meiosis has been observed in *C. albicans*, but tetraploid cells can be induced to reduce their ploidy by concerted chromosome loss [47], which is thought to involve mitotic non-disjunction events and produces diverse aneuploid progeny [48,49,50]. Diversity is the result of (i) cells of differing ploidy or aneuploidy, (ii) the presence of different chromosome homologs (e.g., AA or BB homolog configurations instead of the parental AB configuration), and (iii) extensive recombination between homologs [48,50,51]. Tetraploid cells are also unstable in a murine model of systemic infection and gradually reduce their ploidy over time [40]. In addition, same-sex mating (homothallism) between two **a** cells or between two α cells has been documented under some conditions, and can also lead to the production of diverse parasexual progeny [52,53,54]. 

While >90% clinical isolates are *MTL***a**/α [21,38,55,56], *MTL* homozygous strains can emerge by one of two mechanisms. First, one homolog of Chr 5 can be lost, and the remaining homolog reduplicated. In the laboratory, this can be achieved by growth on an L-sorbose medium which selects for cells that are monosomic for Chr 5. This occurs because multiple negative regulators of the *SOU1* (sorbose utilization) gene which is on Chr 4 reside on the right arm of Chr 5 [57,58,59,60]. Loss of one homolog of Chr 5 therefore leads to the release of *SOU1* repression and permits cell growth on sorbose. Thus, an entire chromosome effectively acts as a single regulatory unit to control growth on this nutrient. Upon removal from sorbose, the single Chr 5 homolog spontaneously reduplicates, generating a disomic, homozygous Chr 5 [58]. A number of clinical isolates are also homozygous for the entirety of Chr 5, indicating that this type of mechanism can occur in nature [20], as well as during laboratory passaging [61]. A second mechanism for homozygosis of the *MTL* locus involves mitotic recombination events that cause LOH at this locus. Analysis of clinical isolates found that several were homozygous only for the *MTL* locus and closely flanking sequences on Chr 5, indicating that gene conversion events that encompassed just this locus were responsible for homozygosis [20]. 

While diploid–tetraploid cycles have been established in *C. albicans*, it was long thought that a haploid state could not exist due to the presence of recessive lethal alleles, and several such alleles have been identified in SC5314 [62,63]. Experiments using strains in which the *RAD52* gene had been deleted suggested that homozygous diploid versions of SC5314 were viable [64]. Subsequently, viable haploid SC5314 cells were uncovered following both antifungal drug treatment in vitro and infection of a murine host [65]. Haploid cells exhibited low fitness and low virulence, and often underwent autodiploidization to generate homozygous diploid cells [65]. Notably, autodiploids also showed lower fitness than heterozygous diploids, establishing that the fitness defect of haploids was due to the loss of heterozygosity and not to decreased ploidy [65].

Aneuploid forms are common products of the parasexual cycle and are also observed in collections of clinical isolates. In the 182 isolates sequenced by Ropars et al., 9.9% were aneuploid, which included both segmental and whole chromosomal aneuploidies [21]. In contrast, Hirakawa et al. found that 8 out of 21 sequenced clinical isolates (38%) were aneuploid [20]. The difference between the two collections may be the result of their exposure to antifungal drugs or other environmental pressures which can select for aneuploid forms [66,67,68,69]. 

The impact of aneuploidy on *C. albicans* phenotypes can be profound and is likely due to changes in gene expression that align with gene dosage. A defining example is that of *C. albicans* cells that acquire an isochromosome 5L (i5L) in response to treatment with fluconazole [66]. Here, the isochromosome provides extra copies of *ERG11* (encoding the drug target) and *TAC1* (encoding a transcriptional regulator of drug efflux pumps) that are the major causes of increased drug resistance [70]. The centromere of Chr 5 contains a long-inverted repeat which promotes recombination and enables the formation of the isochromosome [66]. Furthermore, direct observations reveal that *C. albicans* cells treated with azoles can form tetraploid cells that then undergo aberrant chromosome segregation events to produce aneuploid forms [71]. 

Recent experiments suggest that RNA expression levels often, but not always, correlate with gene dosage in aneuploid *C. albicans* strains. Strains monosomic for Chr 5 showed a twofold decrease in the expression of most genes on this chromosome, and yet 9–16% of genes showed dosage compensation and were expressed at the disomic level, depending on the isolate [72]. A similar trend was observed for RNA levels associated with cells carrying a trisomic chimeric Chr 4/7b, where most genes were expressed in line with their copy number, and yet a subset of genes (~25%) showed dosage compensation to the disomic level [72]. These results extend other studies that indicate close links between chromosome copy number and RNA expression levels in different *C. albicans* lineages [73,74]. 

The reader is also directed to several other recent reviews that discuss how ploidy change and aneuploidy enable adaptation both in *C. albicans* and in other fungal pathogens [75,76,77]. 

## 4. Hemizygosity and Haploinsufficiency in *C. albicans*

**Figure 2:**
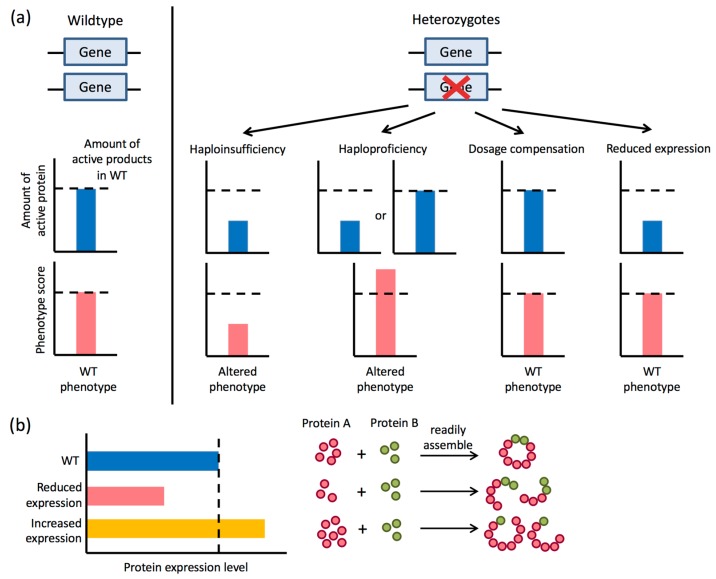
Potential phenotypic outcomes in diploid heterozygotes. (**a**) Loss of one allele of a gene will result in alternative outcomes including haploinsufficiency, haploproficiency and dosage compensation. Haploinsufficiency causes fitness defects, which often constitute a recessive phenotype associated with reduced gene expression. Haploproficiency occurs when one functional allele generates the opposite phenotype of the null mutant, and can be due to either increased or decreased protein levels. Heterozygous cells can also show a normal phenotype, either because reduced gene expression does not produce a phenotype or due to dosage compensation. (**b**) Changes in gene expression are particularly likely to impact phenotypes when the gene product is part of a larger protein complex, as this can cause an imbalance in subunit stoichiometry. WT, wildtype.

The diploid nature of *C. albicans* has historically hindered the targeting of genes for deletion, yet multiple studies have shown that haploinsufficiency can be used to dissect gene function. These studies rely on the fact that strains engineered to lose one functional copy of a gene (i.e., are heterozygous) can produce a noticeable phenotype compared to the parental strain (Figure 2). This approach was adopted by Uhl et al., who performed the first transposon mutagenesis screen in *C. albicans* and identified 146 haploinsufficient genes regulating the transition between yeast and filamentous forms [78]. These results mirrored earlier studies where strains heterozygous for different genes displayed haploinsufficient phenotypes [79,80]. A subsequent study by Oh et al. constructed a genome-wide collection of 3633 transposon mutant strains covering ~60% of the genome and identified 269 genes that were haploinsufficient for growth in one of four conditions [81]. An independent approach undertaken by Xu et al. was to examine 2868 heterozygous deletion mutants for chemically-induced haploinsufficiency and established that this method can uncover the mechanism of action of novel antifungal agents [82]. Chailott et al. similarly used a heterozygous deletion collection consisting of 5470 mutants (representing 90% of *C. albicans* open reading frames) and identified 685 genes that influence cell size [83]. Together, these studies exemplify how haploinsufficiency can be a powerful tool for genome-wide and unbiased screens to dissect multiple *C. albicans* traits. 

Haploinsufficiency has been extensively used by Krysan and colleagues to quantitate genetic interactions in *C. albicans* [84,85,86,87,88]. This includes the examination of strains both for simple haploinsufficiency (observable phenotypes in strains heterozygous for a single gene) and for complex haploinsufficiency (phenotypes in strains heterozygous for two different genes). In complex haploinsufficiency, or CHI, two genes are said to have a genetic interaction if the phenotype of the double heterozygote is more severe than either of the single heterozygotes. These approaches were used to examine the RAM (Regulation of Ace2 and Morphogenesis) signaling pathway that controls filamentation in *C. albicans*. A transposon mutagenesis library was constructed in strains heterozygous for *CBK1,* which encodes a protein kinase central to RAM signaling, and screening identified genetic interactions between the RAM pathway and the PKA-cAMP pathway, as well as with multiple Ace2 targets [84,85]. 

A similar approach was used to examine a set of 133 heterozygous transcription factor (TF) deletion strains for filamentous growth, drug resistance, caffeine sensitivity and virulence. Within this set, haploinsufficiency or haploproficiency was rare, although 10% (13 out of 133) heterozygous mutants exhibited differences in filamentation relative to the wildtype [86]. The competitive fitness of heterozygotes was also examined using a murine model of disseminated candidiasis and 12 mutants from the collection had either decreased fitness (eight mutants) or increased fitness (four mutants), including several genes that impact adherence in vitro [86].

Haploinsufficiency analysis was also applied to the six master TFs in the biofilm network (Brg1, Bcr1, Efg1, Ndt80, Rob1, Tec1; [89]) and revealed that heterozygosity of any of the six factors resulted in a decrease in biofilm formation [87,88]. CHI analysis of the biofilm TFs further revealed that double heterozygote mutants were often as defective as single homozygous mutants in biofilm formation, indicating that the biofilm network is highly efficient but not genetically robust [87]. Significant decreases in biofilm formation were observed even though heterozygous strains expressed five of the six master TFs at ~50% of wildtype levels [87]. This result highlights how small changes in gene expression can have notable effects on *C. albicans* phenotypes. 

It is also interesting that these experiments identified a haploproficient phenotype in an *NDT80* heterozygous strain. Thus, *ndt80/NDT80* cells surprisingly were hyperfilamentous and showed increased biofilm formation relative to the wildtype, despite an *ndt80* null mutant being defective in both of these processes [87]. The *NDT80* heterozygote also showed normal expression levels of the other five master TFs, and yet the haploproficient phenotype was strictly dependent on the presence of two of these TFs, Tec1 and Rob1 [87]. The mechanism underlying the haploproficient *ndt80/NDT80* phenotype is unknown, although a twofold decrease in *NDT80* expression may trigger a compensatory process that stimulates filamentation but is unavailable to strains lacking both *NDT80* alleles [86].

## 5. LOH Is Frequent in *C. albicans*

LOH is essentially an irreversible process by which information is lost from one of the two chromosome homologs. LOH can occur by a variety of processes including chromosome loss/truncation or recombination events that impact short- or long-range segments of the genome (Figure 1). These events have been extensively examined in *C. albicans*, which reveals that LOH rates are elevated in vitro in response to heat stress, oxidative stress, antifungal stress and UV treatment, and that the type of LOH event is impacted both by the stress encountered and by which components of the homologous recombination machinery are present [64,90,91,92].

Genomic changes were examined using experimental evolution experiments in which *C. albicans* strains were resequenced following passage in vitro or following recovery from commensal or systemic models of murine infection [67]. Here, large LOH tracts (and aneuploidy) were occasionally observed, yet short-track LOH events were far more frequent, so that the median LOH size was ~368 bp and impacted only 1–2 heterozygous positions. Strains passaged in vivo showed 11-fold higher mutation frequencies than those passaged in vitro [67], in line with studies that showed higher rates of genomic variation during systemic infection than during in vitro passage [93]. 

Experiments have also examined genetic changes during a murine model of oropharyngeal candidiasis (OPC), and found levels of variation that were two orders of magnitude higher than those in vitro [94]. These included various LOH events and aneuploidies, as well as the emergence of haploid and tetraploid forms, further establishing how host niches can drive genetic adaptation. 

An analysis of oral *C. albicans* isolates from healthy volunteers found evidence for substantial diversity between isolates from a single host [95]. Similar to laboratory microevolution experiments, differences between an individual’s isolates involved numerous short-tract LOH events, with 95% of all LOH tracts having a minimum size of <3 kb. Although rarer, large LOH events were also observed between isolates that impacted hundreds of thousands of SNP positions. Overall, the average number of LOH events between isolates from a single host was 106–254, depending on the carrier [95]. This study establishes that LOH events, particularly short-tract gene conversion events, are a key driver of diversity in *C. albicans* strains in nature. LOH was also frequently observed in a longitudinal set of isolates recovered from oral candidiasis patients treated with fluconazole [69]. Here, LOH events, and not aneuploidy, were associated with the emergence of drug resistance in these isolates, although the exact mechanisms of resistance were not determined. The authors speculate that aneuploid forms could represent key intermediates, leading to more stable genotypes that exhibit drug resistance. 

Interestingly, it was recently shown that many long-range LOH events (and all segmental aneuploidies) detected in a set of *C. albicans* isolates had occurred at long repeat sequences in the genome [96]. For example, 61/153 LOH breakpoints were found within 2 kb of long repeat sequences across all chromosomes. Most of these LOH events were those that extended to the proximal telomere, consistent with a BIR or mitotic crossover event. The *C. albicans* genome contains hundreds of long repeat sequences (in addition to those in repeat gene families or retrotransposons), and the repair of DNA double-strand breaks occurring at these repeats may be the cause of segmental amplifications, deletions, LOH events and inversions in *C. albicans* [96]. 

## 6. LOH Events Are Linked to Phenotypic Change and Host Adaptation

**Figure 3:**
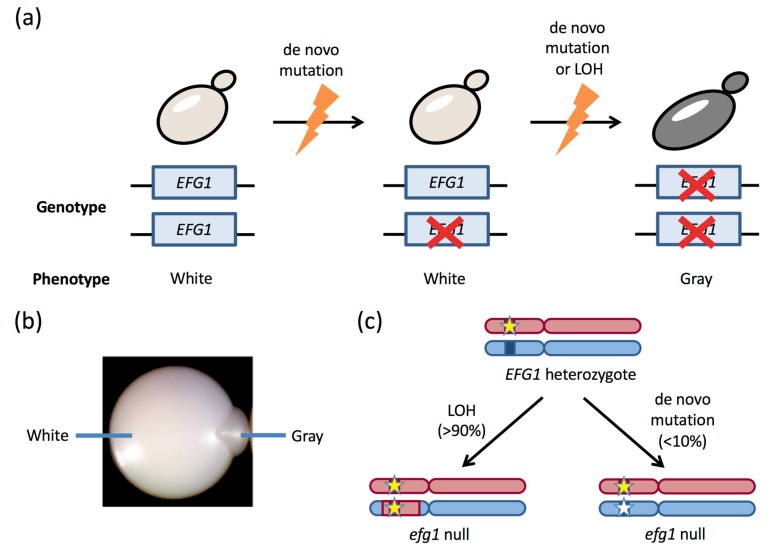
Genetic events at the *EFG1* locus can impact *C. albicans* phenotypes. (**a**) Most *C. albicans* isolates are diploid and carry two functional alleles of *EFG1.* However, one *EFG1* allele can be disrupted by a de novo mutation, and the second allele can then be lost either by LOH or by a second de novo mutational event. Cells with functional *EFG1* are in the ‘white’ state, whereas the complete loss of *EFG1* function causes cells to adopt the ‘gray’ state. (**b**) Image of a single colony showing white *efg1/EFG1* cells that have given rise to a sector of gray *efg1* null cells. (**c**) Clinical isolates that are heterozygous for *EFG1* can lose the functional *EFG1* allele either by LOH (>90% of events) or de novo mutation (<10% of events). Asterisks indicate nonfunctional alleles.

Recent studies have revealed that LOH events are frequently responsible for driving a phenotypic change in *C. albicans* cells. Cells from certain clinical strains had been observed to undergo switching to a ‘gray’ phenotypic state [97], yet it was unknown why only some isolates could adopt this state or the cause of the alternative phenotype. Liang et al. revealed that a subset of clinical isolates is functionally heterozygous for the *EFG1* gene due to ORF-disrupting mutations in one allele. Moreover, isolates that are *efg1/EFG1* heterozygous can stochastically lose the functional *EFG1* allele by LOH (or, on occasion, by de novo mutation), and this causes white cells to transition to the gray state (Figure 3). Gray cells were therefore revealed to be *efg1* null cells [98], and these experiments established that the phenotypic change is due to a high-frequency and specific alteration at the *EFG1* locus. It was also noted that the vast majority of LOH events at *EFG1* involved short gene conversion tracts [98], in line with the sequencing experiments described above that detected multiple short-tract LOH events arising within the *C. albicans* genome [67,95].

*EFG1* encodes a TF that plays a pleiotropic role in *C. albicans* biology, and is known to influence both the commensal and pathogenic properties of the species [80,99,100,101,102]. Notably, it was shown that white *efg1/EFG1* cells inoculated into a murine gastrointestinal (GI) model converted to the gray state after several days, and the emergent gray cells then swept the GI population. Analysis of these gray cells confirmed that they were *efg1* null cells that had lost *EFG1* function, and that this again involved short-tract gene conversion events at this locus [98]. These results extended previous observations that *efg1* null cells exhibit a fitness advantage in GI colonization [99,100,101], and showed how LOH at this locus can drive rapid adaptation in vivo. 

Tso et al. similarly observed loss of function of key genes during microevolution of SC5314 cells in the murine GI tract. Here, the function of *both* alleles of certain filamentation-regulating TFs was repeatedly lost during commensal colonization [103]. *FLO8* was the most frequently mutated TF, although homozygous *efg1* null mutants were also recovered in independent experiments. Examination of *FLO8* alleles revealed that many were homozygous mutants, indicating that they had first acquired a disruptive mutation in one allele that had then undergone LOH [103]. Loss of filamentation (or loss of genes induced during filamentation) therefore appears to be repeatedly selected for in the GI niche. This is in accordance with studies where libraries of TF mutants were screened and filamentation-defective strains (including *efg1* null mutants) were found to exhibit a significant fitness advantage in the GI, whereas mutants that exhibited hyperfilamentous growth were defective for the colonization of this niche [104,105].

A further example where a mutant phenotype has repeatedly emerged in *C. albicans* populations was identified in cystic fibrosis patients. Here, a subset of patients harbored hyperfilamentous *C. albicans* isolates in their sputum, and in the majority of cases this was linked to homozygous mutations in *NRG1* [106], a TF gene that is a negative regulator of filamentation [107]. Different homozygous *nrg1* null mutations were identified between isolates, indicating that loss of *NRG1* occurred independently in different patients and is a common adaptation to the lung environment in individuals with cystic fibrosis [106]. 

## 7. LOH and Drug Resistance in *C. albicans*

A number of studies have established a close connection between gene dosage and drug resistance in *C. albicans*. As discussed above, aneuploid strains (including those carrying an extra i5L chromosome) can exhibit azole resistance due to the increased copy number of genes mediating drug resistance. Moreover, numerous cases have been uncovered where a mutation in one allele causes a certain level of resistance and increased resistance is observed when both alleles are mutated. Thus, mutations in *MRR1*, *TAC1*, *UPC2* and *ERG11* confer resistance to fluconazole when a single allele is mutated, and drug resistance is further potentiated when both alleles contain the same mutation [108]. Here, *ERG11* encodes the direct target of fluconazole, while the other three genes encode transcription factors that regulate expression of drug efflux pumps or *ERG11* itself [109,110,111,112,113,114,115,116,117]. Analysis of allelic configurations are relevant, as patient isolates are often homozygous for drug resistance mutations, and some of these mutations are recessive so that resistance is only evident following LOH [110,113,114,116,118,119]. Mapping of LOH events at these genes reveals that homozygous is often due to mitotic recombination (gene conversion or single crossover/BIR events), although chromosome loss and reduplication is also observed [113,114,116,120]. Furthermore, inspection of *C. albicans* strains has revealed that changes in chromosome copy number and mitotic recombination can both occur in a single isolate and can act in combination to elevate drug resistance [114,120].

LOH of the *MTL* locus has frequently been found to have occurred in fluconazole-resistant isolates [121]. This is due, at least in part, to this locus being located on the left arm of Chr 5, the same arm as *TAC1* and *ERG11* genes. LOH events at *TAC1* or *ERG11* may therefore extend to the flanking *MTL* locus. Comparison of *C. albicans* strains with different *MTL* configurations found that *MTL* homozygous strains were not inherently more drug resistant than *MTL* heterozygous strains [55,122]. However, a subsequent study showed that LOH of the *PAP1* gene residing within the *MTL* locus could affect resistance. This is because in the absence of *PAP1***a**, the *PAP1*α allele causes hyperadenylation and increased stability of *CDR1* transcripts that encode for a multidrug transporter that can potentiate drug resistance [123]. 

A recent study showed that linkage between the *MTL* and key drug resistance genes may also enable the spread of resistance mutations within a population. In this scenario, if a heterozygous resistance mutation arises in *TAC1* or *ERG11,* then LOH at these loci will further increase the level of resistance. If LOH events include the linked *MTL* locus, the resultant *MTL* homozygous cells will be competent to mate, and can generate recombinant products that have combined resistance mutations from different lineages [120,124]. This mechanism was demonstrated to generate highly drug-resistant cells under laboratory conditions, establishing that mating can potentially accelerate the emergence of drug-resistant variants of *C. albicans* [124].

## 8. Aneuploidy and LOH during Strain Construction in *C. albicans*

Studies have revealed that *C. albicans* genomes frequently undergo rearrangements during genetic manipulation and that these changes can impact phenotypic traits. Observed genomic changes involve a wide variety of aneuploid forms, including amplification of chromosomes, loss of whole chromosomes, or the truncation of chromosome arms [73,74,125,126,127]. Moreover, these changes can occur at chromosomes other than those being targeted for genetic manipulation [73]. 

In addition to aneuploid forms, unintended LOH events have frequently arisen in laboratory lineages that can affect *C. albicans* traits. For example, Abbey et al. showed that large LOH tracts were detected in strains derived from SC5314 and again impacted multiple chromosomes [128]. The largest of these emergent LOH tracts was 1330 kb on the left arm of Chr 2. In addition, extensive LOH led to decreased overall levels of heterozygosity that correlated with longer cell doubling times, indicating that these events impacted overall cell fitness [128].

Further examples of unintended LOH events include homozygosis of Chr R during the construction of a *sap4 sap5 sap6* triple null mutant. Critically, as a result of the homozygosis event, loss of the unlinked *SAP2-2* allele occurred in this strain background. The consequence is that while it was initially believed that loss of Saps 4–6 led to a growth defect when proteins were the sole nitrogen source [129], this phenotype was subsequently found to be attributable to loss of the *SAP2-2* allele [130]. Loss of the single *SAP2-2* allele shows a strong phenotypic effect as the product of this allele is required for full induction of both *SAP2* alleles [26].

A recent phenotypic trait attributed to a spontaneous LOH event involves sensitivity to the DNA-damaging agent methyl methane sulfonate (MMS). Here, homozygosis of a region on the right arm of Chr 3 was found to be responsible for increased sensitivity to MMS in CAI4, a lineage derived from SC5314 [131]. The causative gene was *MBP1,* as strains lacking one *MBP1* allele were MMS sensitive, whereas strains lacking the other allele were not, and resistance was restored to susceptible strains by reintegration of the more active *MBP1* allele [131]. All of the examples listed here highlight the need for diligence and a careful interpretation of results in genetic studies of *C. albicans*.

## 9. Conclusions

The genome of *C. albicans* exhibits a remarkable degree of plasticity due to a wide variety of mutational processes. These can affect gene dosage via changes in ploidy, aneuploidy or copy number variation, or can alter allelic configurations including those impacting mating and the *MTL* locus. Genetic changes have been shown to drive adaptation due to loss of filamentation in the GI tract or via hyperfilamentation in the lungs of cystic fibrosis patients, and are also critical to the emergence of drug resistance in the clinic. In addition, haploinsufficiency has emerged as a powerful approach for the identification and analysis of gene function in this diploid species. With advances in sequencing approaches, the understanding of the *C. albicans* traits affected by genomic alterations will accelerate. For example, recent findings in *S. cerevisiae* reveal how certain heterozygous mutations can increase genome instability in a manner that parallels that in cancer cells [132] and that even missense or ‘silent’ synonymous changes can make important contributions to the phenotypes of isolates [133]. It is therefore envisaged that understanding of the mechanisms and consequences of genome plasticity in *C. albicans* will continue and will provide new insights into this important commensal and pathogen.
